# Supplementary figures and images for: An approximate full-likelihood method for inferring selection and allele frequency trajectories from DNA sequence data
Source: PLoS Genet. 2019 Sep 13;15(9):e1008384. doi: 10.1371/journal.pgen.1008384 (PMC6760815; doi:10.1371/journal.pgen.1008384)

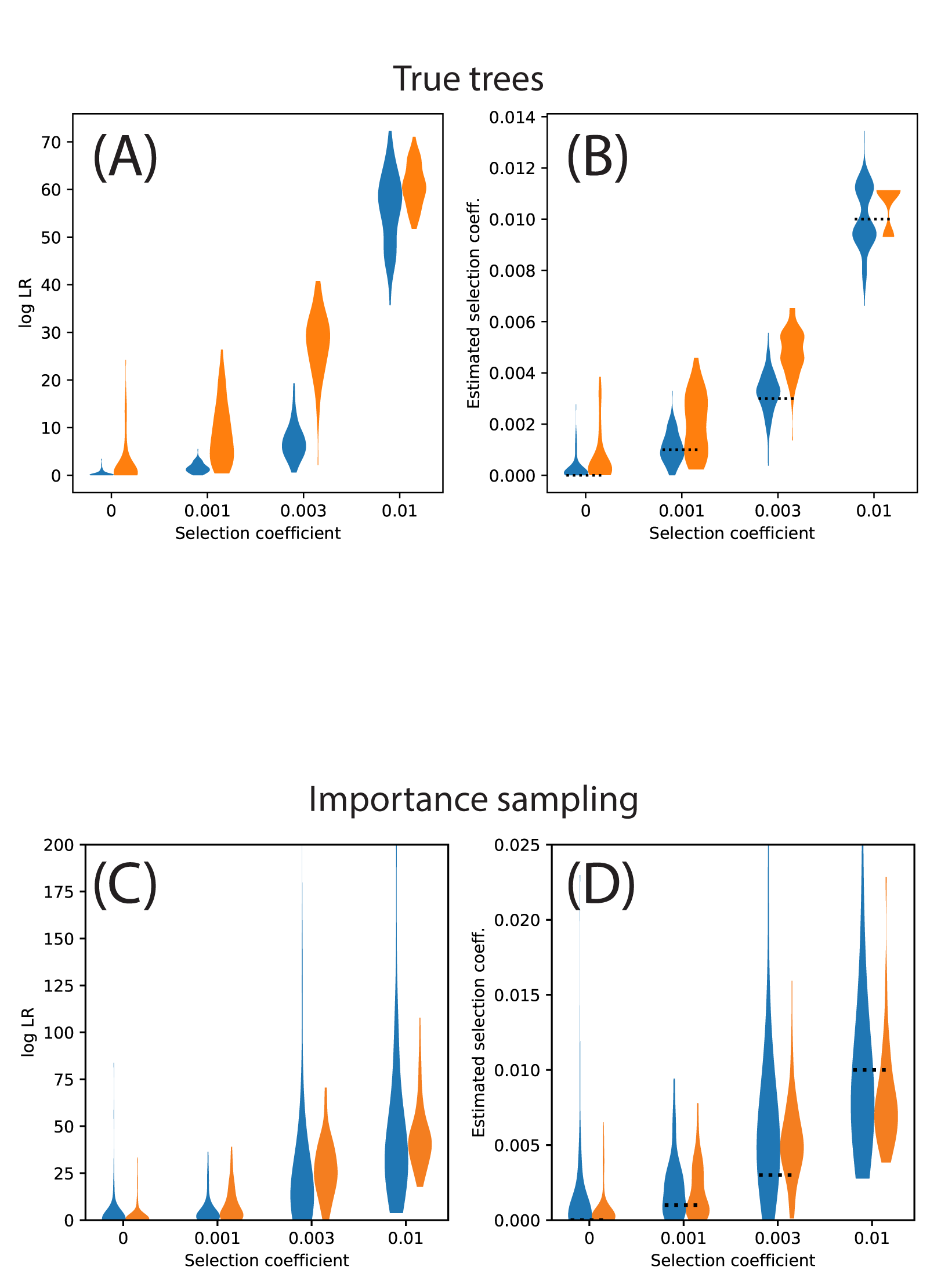

Supplement: S9 Fig — Likelihood ratios (A,C) and selection coefficient estimates (B,D) calculated given the true tree (A,B) and via importance sampling using ARGweaver (C,D). Blue violin plots represent estimates using the correct (European) demographic model, whereas orange plots represent estimates using a model of constant Ne = 104. Simulations were done under the European demographic model described in Methods and Materials using a locus of 200kb, n = 25 diploid individuals and μ = 2.5 × 10^−8^ mut/bp/gen, r = 1.25 × 10^−8^ recombinations/bp/gen. (TIF) [file pgen.1008384.s009.tif]
